# Case report: Professional cyclist diagnosed osteoarthritis of the knee grade II

**DOI:** 10.1002/ccr3.3555

**Published:** 2020-11-20

**Authors:** Jorge Velázquez‐Saornil, Angélica Campón Chekroun, Elena Sánchez Jiménez, Ana Martín Jiménez, Manuel Vicente García, Sonia Gómez Sánchez, Encarnación Méndez Sánchez, Zacarías Sánchez Milá

**Affiliations:** ^1^ Universidad Católica de Ávila Ávila Spain; ^2^ FisioSalud Ávila Ávila Spain

**Keywords:** biomechanics, osteoarthritides, knee, physiotherapy techniques, sport medicine

## Abstract

After a physical therapy treatment on the injured knee and a biomechanical study of the position on the bicycle, the symptomatology of the patient's injured knee has improved.

## Graphical abstract

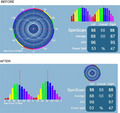

## 1 INTRODUCTION

Osteoarthritis (OA) is a degenerative and chronic disease affecting the joints of the human body. This disease consists of progressive wear and tear of the cartilage, although it also affects other joint structures.^1^

The pain produced by OA of the knee is more intense during a period of movement, and this diminishes when there is rest. In cases where knee OA is more severe, the pain occurs even at rest.^2^

From an anatomical point of view, the knee is one of the most important joints in the human body, as it is involved in most human movements; it is also a joint that bears heavy loads and high stress, making it susceptible to injury more frequently than other joints.^3^, ^4^

Among the risk factors for this ailment are overweight, age, gender, type of work, old injuries, bone mineral density, genetic factors, nutrition, and lifestyles. Each of the above factors contributes differently to disease progression.^5^, ^6^

## 2 OSTEOARTHRITIS OF THE KNEE

Classification of osteoarthritis kellgren and lawrence.^7^

GRADE 0: no radiological alterations.

GRADE 1: doubtful narrowing of the articular space. Possible osteophytosis.

GRADE 2: possible narrowing of the articular space. Osteophytosis.

GRADE 3: narrowing of the articular space. Moderate osteophytes. Slight sclerosis. Possible deformity of the ends of the bones.

GRADE 4: Marked narrowing of the articular space. Abundant osteophytosis. Severe sclerosis. Deformity of the ends of the bones.

## 3 OBJECTIVES

- To develop a treatment plan for a knee OA patient based on the scientific literature.
- To determine the most effective treatment for the pathology studied.
- To check the proposed treatment if it is effective to pain, functionality and rigidity, quality of life.

## 4 MATERIAL AND METHODS

### 4.1 Anamnesis

Professional cyclist (29 years old), apart from the hours of cycling training, he runs a continuous 2 hours a week on a treadmill, strengthening both the upper and lower train muscles. He presents with progressive pain in his right knee; this pain has progressively increased with the passage of time in training. The pain prevents him from preparing competitions correctly and with a guarantee. BMI: 20.3.^8^

### 4.2 History current pathology

- Pain in the knee joint, pain is concentrated in the area of the joint, pain is greater the burden of training.
- Pain in your lower back and middle back.
- He points out that he currently sleeps more hours than in previous months, and there has been no change in any activity or behavior.
- Refers to increased general fatigue.

As for the diet is correct and varied, he eats a large amount of fruit and that he makes the five meals a day.

Before consulting, the patient has taken nonsteroidal anti‐inflammatory drugs (NSAIDs) to reduce pain; these were effective during the time of action, but have not been able to eliminate the discomfort. He has performed knee muscle stretching and cryotherapy twice a day for 10 minutes each exercise.

### 4.3 Medical treatment

Cryotherapy and NSAIDs have been used previously.

With respect to the use of medication and treatment products, substances prohibited by the International Cycling Union (ICU) or the World Anti‐Doping Agency (WADA) may not be used. In the event of having to use any of the substances banned by either of the two bodies mentioned above, this should be communicated and approval should be given for the use of the substance.

### 4.4 Physiotherapy intervention plan

The treatment must be as effective as possible to achieve a recovery as quickly as possible. Objectives:

- Eliminate pain from the area.
- Achieve the entire ROM.
- Restore the patient's normal function and activity.

The treatment is 20 sessions, 5 days a week during the first part of the treatment and alternating days during the second phase. The first 8 sessions will be shortened, in which the treatment focuses more on analgesia and joint range.

During this phase, which lasts eight sessions, this treatment will be repeated daily.

- TENS: 20 min. The electrodes 25 mm by 25 mm electrodes. The parameters were pulse 150 ms., frequency 150 Hz.^9^, ^10^
- Ultrasound 1 W/cm^2^ continuous, 9 minutes application.^11^, ^12^, ^13^
- Passive stretching of quadriceps, hamstrings, and adductors. There are 3 series of 10 repetitions with 15 seconds of stretching.^14^
- Cryotherapy for 10 minutes, with an ice pack applied to the area.^15^
- Kinesiotapping. The kinesiotape is applied on fridays to reduce the pain on knee.^16^

The second phase has a duration of 12 alternate sessions. At the end, the patient is re‐evaluated and if the result is optimal, the patient is discharged.^5^

- Ultrasound 1 W/cm^2^, during 9 minutes continuous mode.
- Strengthening of the musculature. Kotz electrotherapy. For quadriceps and hamstrings.^10^
  - 2 series of 20 minutes each series and 5 minutes break between series and series.
  - 2500 Hz
  - 50 Hz AMF.
  - Stimulus ("1/5/1/20") (Ascent ramp/ plateau/ descent ramp/rest)
- Passive stretching of quadriceps, hamstrings, and adductors. There are 3 series of 10 repetitions with 15 seconds of stretching.^10^
- Cryotherapy for 10 minutes, with a cold pack applied to the area.^17^
- Kinesiotape for knee OA.^16^

### 4.5 Complementary tests

#### 4.5.1 Magnetic resonance

Magnetic resonance imaging (MRI) is the complementary test that allows a more accurate assessment as it allows both soft tissue and bone tissue to be observed with high resolution in several planes.^18^

The patient undergoes an MRI scan to check for knee injuries. The study shows a grade 3 osteoarthritis that is accompanied by narrowing of the joint space and mild bone sclerosis, in addition to a slight joint effusion.

#### 4.5.2 Biomechanical study

Biomechanical studies are increasingly used by athletes to prevent injuries and improve sports performance. These studies are mainly used in cycling and athletics. For its use in cycling, a specific material is needed that is formed by a set of cameras and sensors, which analyze the posture of the cyclist at first; from this first data collection, a specialized software makes an analysis of them and gives some guidelines to correct the posture. To complement this biomechanical study, a cycloergometer is sometimes used. The cycloergometer is a device that is used to analyze the pedal in depth, to see the dead points of the same one and thus to be able to see the losses of power in the phase of the pedaleo.^19^

The data obtained from this study are that the measurements you take on the bike with which you have done the training are inappropriate. Above all, the height of the seat post, the distance from the handlebars to the saddle, and the position of the cleats can have a negative impact on the patient and present discomfort in the spine and/or knees.

**FIGURE 1:**
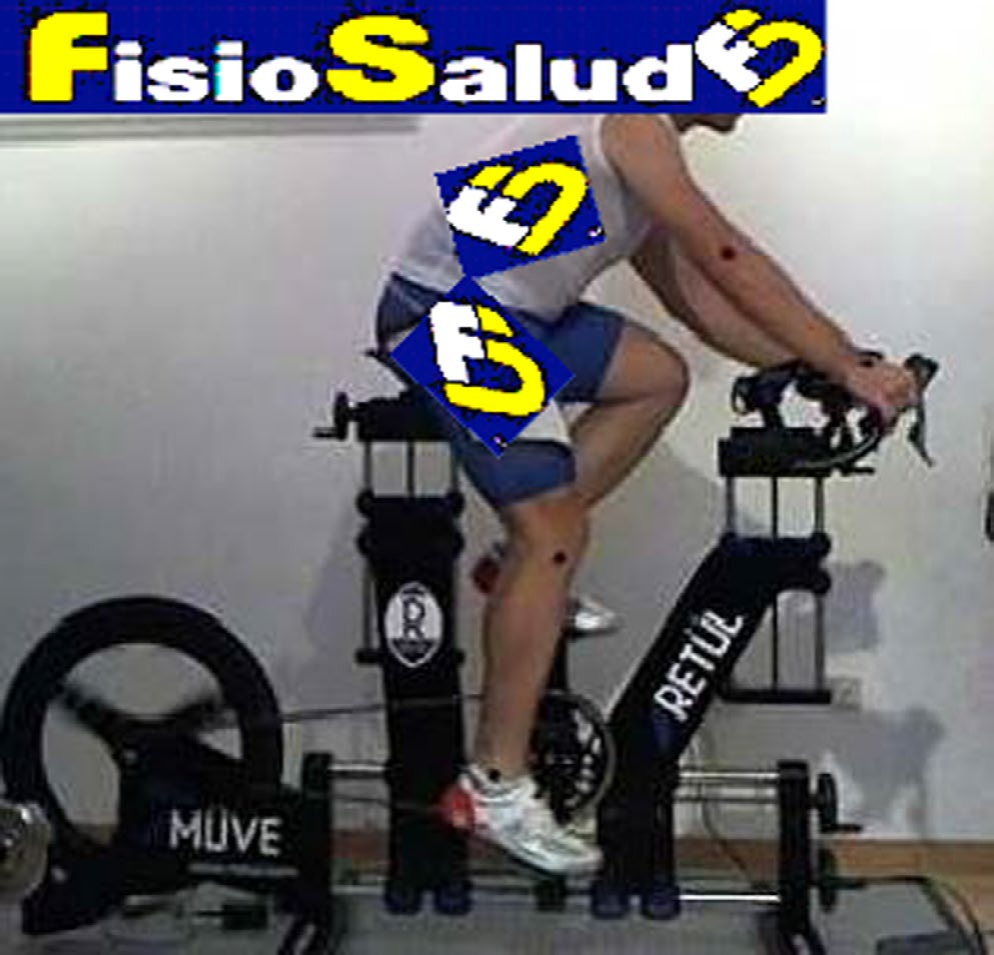
Biomechanic study

**FIGURE 2:**
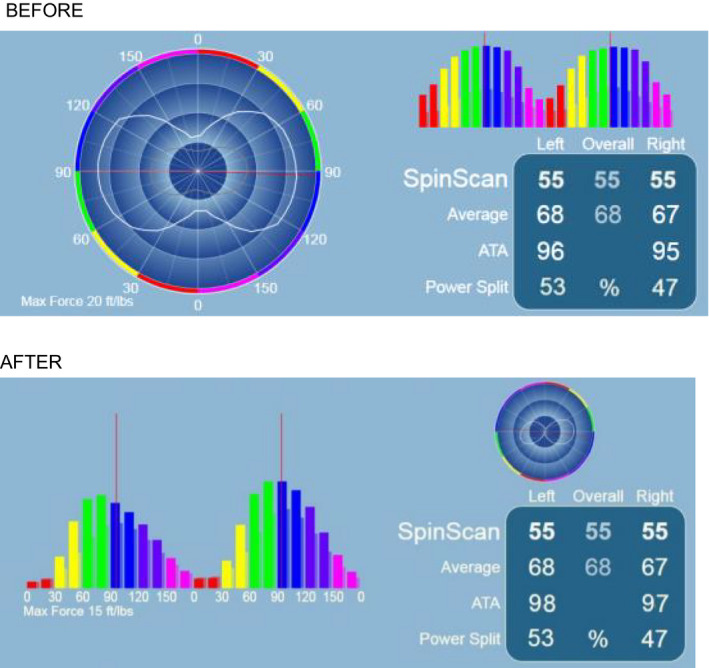
Pedal stroke análisis

**FIGURE 3:**
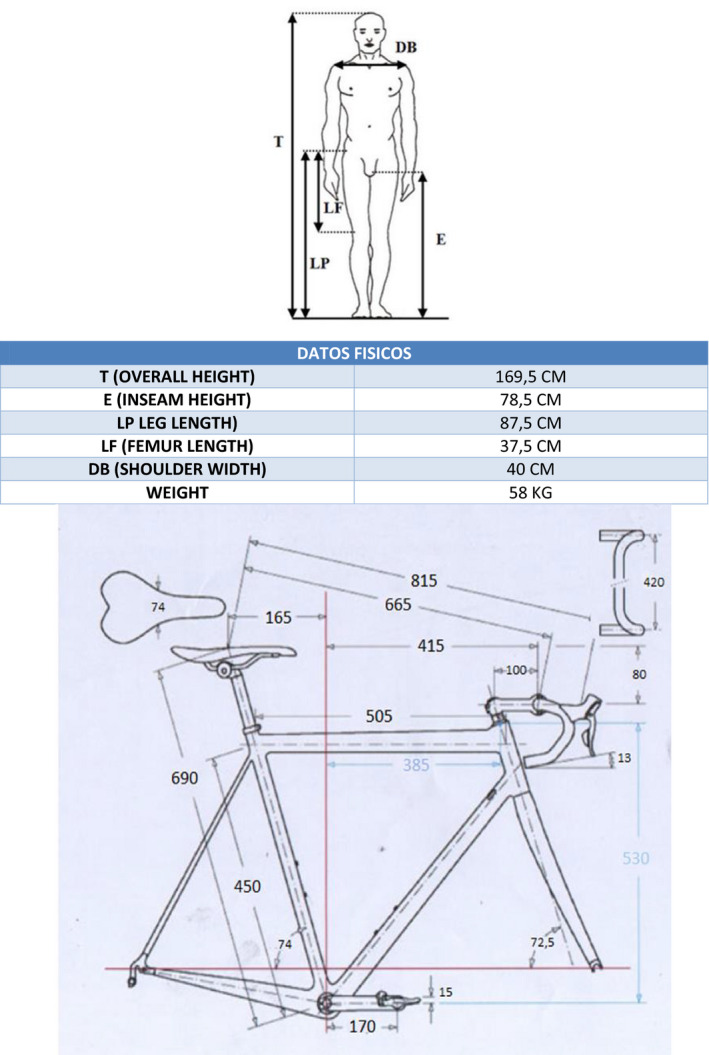
Physical and bicycle measurements

This is achieved by changing the height of the saddle and the distance from the handlebars to the saddle, which in this case is reduced by 9 mm in the first case and reduced by 12 mm in the second, is to reduce the tension in the area of the lumbar spine and in the area of the back of the lower limb. With regard to the modification of the cleats, a delay was made and a small rotation of the cleats on the support surface; the gap was 3 mm and 4° of right rotation for the right cleat; in the left cleat, the delay was 2 mm and the rotation was 7° to the left. The modification of the delay in the cleats has two objectives, the first is that the foot in the ascent phase and the descent of the pedal goes up and down flatter and that the transmission of force is more uniform. All in all, a more effective pedaling and less power loss were achieved, as well as a lower tension in the area of the twin and soleus in the pedaling. With the rotation what is obtained is that the knee; this as centered as possible so that it transmits the power correctly. (Figures 1, 2, 3).

In this study, a power loss test is also performed during the pedaling phase. These measures are carried out before and after the correction of the measures in order to be able to verify the improvement. (Figure 3).

The data obtained from this test are as follows: before the correction, there is a 4% loss of power in the left leg and 5% loss in the left leg. After the adjustments made on the bicycle, the loss percentages are 2% on the left and 3% on the right. (Figure 2).

## 5 RESULTS

It is verified from the different evaluations that carried out the patient, is that the OA knee may be caused by poor posture when performing the exercise. This is one of the reasons why a biomechanical study was carried out, and subsequently, a correction was made to the parameters of the bicycle.

The following sections show the patient's evolution.

### 5.1 Evolution of the EVA scale

**FIGURE 4:**
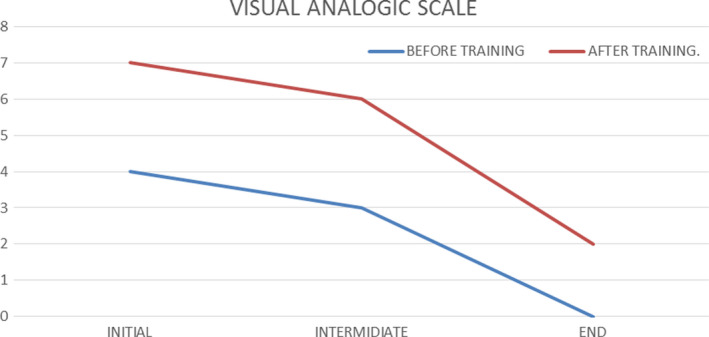
Vas study

First of all has been a decrease in pain using different physiotherapy techniques in addition to an increase in joint range.^20^ This decrease in pain can be seen in the VAS scale as can be seen in the following graph (Figure 4).

### 5.2 Evolution Ipaq scale

**FIGURE 5:**
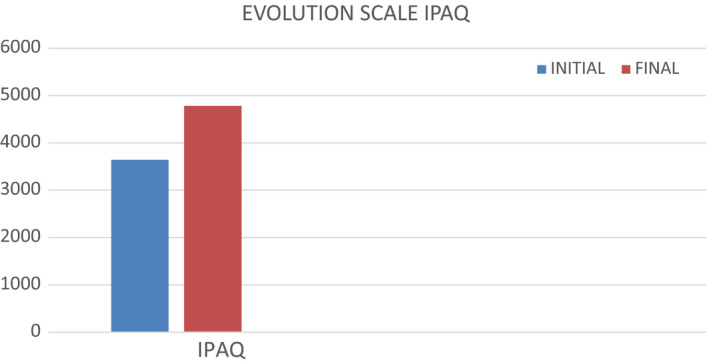
IPAQ scale evolution

The IPAQ scale is used to check the level of physical activity; the graph shows how the level of physical activity has increased (Figure 5).^21^

### 5.3 Womac scale evolution

**FIGURE 6:**
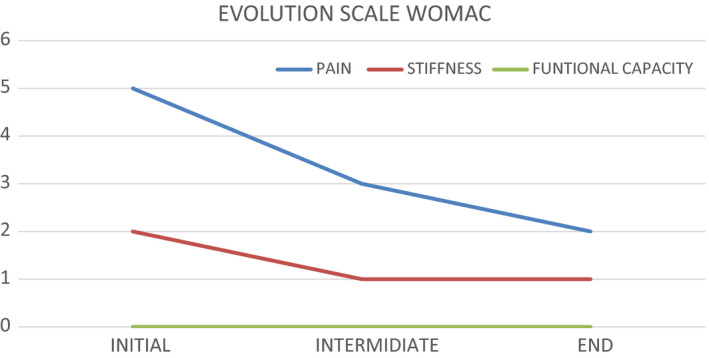
WOMAC scale evolution

The WOMAC scale was carried out in all three assessments, in all of them the assessment of pain and stiffness has decreased while functional capacity has been maintained (Figure 6).^22^

### 5.4 Evolution amplitude of the knee

**FIGURE 7:**
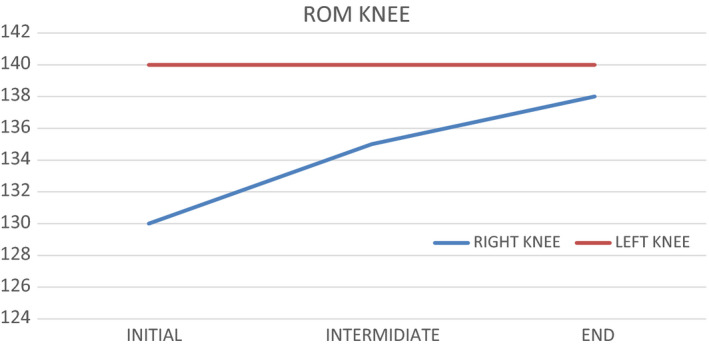
ROM knee

The amplitude of the knee has increased with treatment, and the amplitude of the left knee has almost been reached (Figure 7).^23^

## 6 DISCUSSION

After a physiotherapy treatment in the injured knee,a biomechanical study of the position on the bicycle has improved the patient's symptomatology. It can be seen how by modifying the height of the saddle (descent of 9 mm) and the distance from the handlebars to the saddle (reduced by 12 mm), the tension in the back area and in the area of the posterior muscular chain can be reduced. A modification of the cleats is also carried out with a delay and a small rotation of the cleats on the support surface; the gap was 3 mm, and 4° of right rotation for the right cleat. In the left cove, the delay was 2 mm, and the rotation was 7° to the left. The modification of the delay in the cleats has two objectives, the first is that the foot in the ascent phase and the descent of the pedal goes up and down flatter and that the transmission of force is more uniform. All in all, a more effective pedaling and a lower power loss are achieved, as well as a lower tension in the gastrocnemius and soleus in the pedaling phase. With the rotation what is obtained is that the knee is as centered as possible so that it transmits the power correctly.

In this study, a power loss test is also performed during the pedaling phase (cicloergometer). These measures are carried out before and after the correction of the measures in order to be able to verify the improvement. The data obtained from this test are as follows: before the correction, there is a 4% loss of power in the left lower limb and 5% loss in the right lower limb. After the adjustments made on the bicycle, the loss percentages are 2% on the left and 3% on the right.

It shows how slight changes in the position of the patient during pedaling on the bicycle can modify the biomechanical joint of the patient during exercise. The relevance of these data lies in the repetition of the sporting gesture, since the patient undergoes training and competition for many hours at the end of the day with an elevated pedal cadence and an incorrect position of the patient on the bicycle causes irritation of different joints and muscle overload. By means of biomechanical corrections and an adequate physiotherapy plan, these injuries will alleviate the patient's symptomatology, which means an advance in the prevention of both acute and chronic injuries and a solution to discomfort during professional competition.

### 6.1 Limitations

A reduced sample size and a short‐term follow‐up plan are used, as the patient had to improve quickly when immersed in elite competition.

The biomechanical study was carried out with body sensors and a dynamic analysis of the 3D pedal using the Retül system, recognized worldwide as the best cycling analysis system in terms of precision and experience. Perhaps the results should be checked with another biomechanical measurement system to avoid bias and compare all measurements.

## 7 IMPLICATIONS FOR PHYSIOTHERAPY PRACTICE

The treated clinical case, being an elite athlete participant to strict anti‐doping controls, has conditioned the application of the treatment. From the beginning, a conservative treatment has chosen, with special care in the use of products and substances.

- A treatment plan has been drawn up for a patient with OA of the knee based on scientific literature, taking into account the different types of studies for each of the techniques used in the patient.
- The proposed treatment (ultrasound, tens, cryotherapy, stretching, and kinesiotaping) has been effective in reducing pain, improving function, improving joint range, and physical activity.
- The biomechanical study of the cyclist's position during pedaling and the study of power using a cycloergometer have been of vital importance to avoid knee injuries.

## CONFLICT OF INTEREST

None declared.

## AUTHOR CONTRIBUTIONS

JVS: involved in writing the original draft, formal analysis, review, and editing. ACC: involved in conceptualization, writing the original draft, formal analysis, writing, review, and editing. ESJ: involved in software, writing, review, and editing. AMJ: involved in methodology, writing, review, and editing. MVG: involved in conceptualization, writing the original draft, writing, review, and editing. SGS: involved in software, writing, review, and editing. EMS: involved in software, writing, review and editing. ZSM: involved in conceptualization; writing the original draft, formal analysis, writing, review and editing.

## Data Availability

We declare that the patient has signed the informed consent to carry out this work and has allowed us to collect data and make future publications with this research. Furthermore, this consent reflects the willingness to collect physical and biomechanical images with the possibility of collecting information and its subsequent disclosure. This study has been carried out with the approval of the Ethics Committee and under the Declaration of Helsinki. The data set will be archived for at least 10 years after publication.
